# The effectiveness of the Wim Hof method on cardiac autonomic function, blood pressure, arterial compliance, and different psychological parameters

**DOI:** 10.1038/s41598-023-44902-0

**Published:** 2023-10-16

**Authors:** Sascha Ketelhut, Dario Querciagrossa, Xavier Bisang, Xavier Metry, Eric Borter, Claudio R. Nigg

**Affiliations:** Institute of Sport Science, University of Bern, Bremgartenstrasse 145, 3012 Bern, Switzerland

**Keywords:** Lifestyle modification, Preventive medicine

## Abstract

The Wim Hof method (WHM) is a multi-disciplinary approach to physical and mental well-being combining cold exposure, breathing exercises, and meditation. This study evaluated the effects of a 15 days WHM intervention on cardiovascular parameters at rest and during a cold pressor test (CPT), as well as on various psychological parameters. Forty two participants were randomized into an intervention (IG) and a control group. Throughout the 15 days intervention, the IG performed the WHM daily. Before and after the intervention, systolic (SBP) and diastolic blood pressure (DBP), pulse wave velocity (PWV), heart rate (HR), root mean sum of squared distance (RMSSD), and standard deviation of RR-intervals (SDNN) were assessed at rest and during a CPT. Furthermore, perceived stress (PSS), positive affect (PANAS+), negative affect (PANAS−), and subjective vitality (trait (SVSt) and state (SVSs)) was determined. No significant time × group interactions could be detected in HR (*p* = 0.709); RMSSD (*p* = 0.820), SDNN (*p* = 0.186), SBP (*p* = 0.839), DBP (*p* = 0.318), PWV (*p* = 0.983), PANAS+ (*p* = 0.427), PANAS− (*p* = 0.614), SVSt (*p* = 0.760), SVSs (*p* = 0.366), and PSS (*p* = 0.364). No significant time × group effects could be detected during the CPT (ΔHR: *p* = 0.596; ΔSBP: *p* = 0.366; ΔDBP: *p* = 0.999; ΔPWV: *p* = 0.635; perceived pain: *p* = 0.231). Performing the WHM daily did not exert positive effects on cardiovascular and psychological parameters.

## Introduction

Cardiovascular diseases (CVDs) are a major cause of morbidity and mortality worldwide^1^. In 2019 CVDs accounted for 17.9 million deaths, representing 32% of all deaths across the globe^1^. Despite the availability of established prevention and treatment approaches, CVDs have remained the leading cause of disease burden for decades. Besides pharmacotherapy and lifestyle changes, additional modalities are gaining popularity as treatment approaches. Mindfulness-based interventions^2^, cold water immersion^3^, and breathing exercises^4^ have been discussed as possible approaches to reduce cardiovascular risk and may be used as complementary therapies in cardiovascular prevention.

Mindfulness-based interventions aim to reduce blood pressure (BP) by employing stress-reduction techniques, including meditation, which are designed to foster a state of inner awareness and calmness^5^. A recent meta-analysis by Shi and colleagues^2^ reveals a decrease in systolic BP of up to − 6 mmHg and in diastolic BP of up to − 4 mmHg following various mindfulness-based interventions. Furthermore, Goyal et al.^6^ show that mindfulness meditation programs can lead to moderate reductions in various dimensions of psychological stress.

Cold water immersion is hypothesized to result in increased body sturdiness, inter alia, through long-term antioxidative adaptation^7^, and an increase in several components of the immune system^8^. Furthermore, positive effects of regular cold water immersion on feelings of energy, pain relief, and overall well-being have been documented^9^. Even though acute cold water exposure represents a significant cardiovascular stress stimulus and poses a significant risk of death, particularly for those with underlying structural heart disease, regular cold water exposure has been reported to enhance cardiovascular health through various adaptive mechanisms^3^.

Breathing exercises have garnered considerable attention as promising approaches for both the treatment and prevention of cardiovascular disease. Research has demonstrated that these techniques can contribute to stress reduction^10^, and lower BP^11,12^ underscoring their potential therapeutic impact on cardiovascular health.

The Wim Hof Method (WHM), a multi-disciplinary approach to physical and mental well-being, has recently gained tremendous popularity. Developed by Dutch extreme athlete and multiple world record holder Wim Hof, the WHM combines cold exposure, breathing exercises, and mindfulness meditation. The cold exposure component involves exposing the body to colder temperatures through activities such as ice baths, cold showers, and cold-water immersion. The breathing exercises involve a specific pattern of deep breaths, retention, and exhalation designed to relieve tension, and activate the autonomic nervous system. The mindfulness component involves meditation and visualization techniques aimed at improving focus and reducing stress^13^.

The WHM has been claimed to have a range of benefits, including improved mood, reduced stress and anxiety, enhanced immune function, improved cardiovascular function, and increased energy and vitality, which should be evident after only 10 days^13^. Despite its growing popularity, there is limited scientific evidence to support these health claims.

Preliminary research suggests that the WHM may have benefits in areas such as inflammation and mood regulation. Petraskova et al.^14^ found that an intervention applying the WHM was associated with a reduction in depressive symptoms in members of an Antarctic expedition. Kox et al.^15^ and Zwaag et al.^16^, found that subjects who performed the WHM showed a significantly attenuated inflammatory response compared to a control group during experimental human endotoxemia.

Further research is necessary to understand the effects of the method and its underlying mechanisms. Currently, the effects of the WHM on cardiovascular risk factors have not been assessed.

Beyond BP, which is arguably one of the most robust predictors of future cardiovascular health, other risk markers like pulse wave velocity (PWV) have gained significant attention^17^. PWV characterizes the speed of the central pulse wave and serves as an indicator of arterial stiffness, offering a reliable and non-invasive tool to identify subclinical atherosclerosis and enhance risk assessment for CVDs^17^. Elevated PWV is associated with the development of hypertension and serves as a predictive factor for cardiovascular events, even after accounting for other well-established risk factors^18^. Additionally, parameters of heart rate variability (HRV), which measure the beat-to-beat fluctuations in the time intervals between consecutive heartbeats, offer a sensitive measure of cardiac autonomic control and serve as an early marker for CVDs^19^.

Consequently, the present study aimed to assess the effects of the WHM on BP, PWV, and HRV. Moreover, the possible effects of the WHM on cardiovascular response to a stressor have yet to be assessed. Literature shows that cardiovascular reactivity can predict future cardiovascular morbidity and mortality and is thus regarded as an important risk factor for developing future CVDs^20^.

Research on possible positive effects of the three components (cold exposure, breathing exercise, meditation) of the WHM supports the assumption that this method may induce positive effects on cardiovascular risk factors^2,4,21^. Moreover, these components have the potential to elicit beneficial impacts on one's perceived stress, affect, and vitality^9,22^, which are related to mental and cardiovascular health^23–26^.

In this study, we aim to evaluate the effects of a 15 days WHM intervention on different cardiovascular parameters at rest and during a standardized stress test, as well as on various psychological parameters to better understand its potential benefits and limitations.

## Methods

### Study design and participants

**Figure 1:**
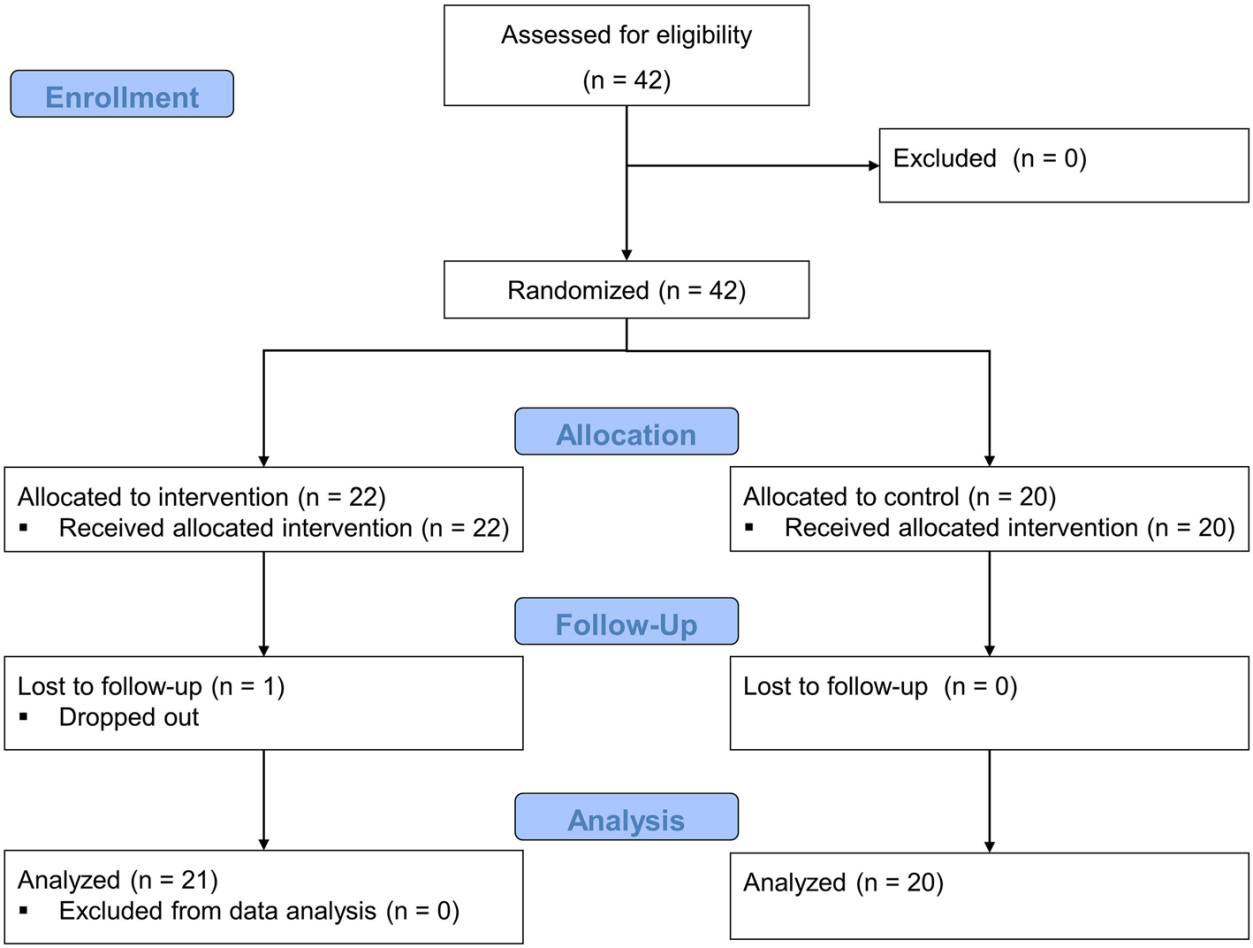
Flow-chart.

This study was designed as a randomized controlled trial with parallel arms. Participants were recruited between May and July 2022 through social media and personal contacts. Eligibility criteria included (1) being male, (2) having no underlying health conditions, (3) not using antihypertensive or other cardiovascular medications, (4) not engaging in regular exercise training, (5) and not having prior experience with any of the components of the intervention (breathing, meditation, or cold exposure). A total of 42 participants (aged 26.7 ± 5.7 years; body mass index 22.8 ± 2.0 kg/m^2^) volunteered to participate in the study (Fig. 1). All participants received a verbal and written explanation of the study´s objective and procedures and gave written informed consent. The study was conducted in accordance with the Declaration of Helsinki, and approved by the Ethical Commission of the Faculty of Human Sciences University of Bern (Nr. 2022-04-00004). The study was registered on ClinicalTrials.gov (https://clinicaltrials.gov/ct2/show/NCT05894031, date 08/06/2023).

All measurements were performed in a controlled environment at the Physiology Lab of the Institute of Sports Science at the University of Bern. The measurements were carried out by trained study staff under controlled conditions using the same equipment and procedure. The participants visited the lab on two occasions for baseline and follow-up measurements, with both sessions taking place at similar times of the day to rule out circadian influences on outcomes. The participants were instructed to visit the lab at least 4 h postprandial and to avoid consuming caffeinated or alcoholic beverages and nicotine for 4 h prior to the measurements. They were further advised to abstain from vigorous physical activity for at least 24 h before each test day.

The study protocol included the administration of questionnaires to obtain information on the participants’ demographics, medical history, habitual physical activity, perceived stress, affect, and vitality. Subsequently, heart rate (HR), HRV and BP readings were conducted. Finally, a cold pressor test was performed to assess HR, BP responses, and perceived pain.

After baseline assessments, the participants were randomly allocated to either the intervention group (IG) or the control group (CG) by the principal investigator using a computer-generated random number table. The IG participated in the WHM daily for 15 days while the CG continued with their normal daily activities. During the intervention period, participants were asked to maintain their usual diet and physical activity patterns.

### Measurements

#### Anthropometrics

Height and body mass were assessed using a stadiometer and scale (BC-545 Innerscan, Tanita, Netherlands). Waist circumference was measured to the nearest 0.1 cm at the midpoint between the iliac crest and the lowest ribs. Body Mass Index (BMI) was calculated as body mass in kilograms divided by height in meters squared (kg/m^2^), and Waist-to-Height Ratio (WHtR) was determined as the ratio of waist circumference to height (waist circumference/height).

#### Physical activity

Self-reported physical activity was assessed using a modified version of the Godin-Shephard Leisure-Time Physical Activity Questionnaire,a  valid and reliable tool for assessing physical activity^27,28^. The German version^29^ of this questionnaire was employed and the metabolic equivalent of task (MET) hours were derived from this questionnaire.

#### Heart rate variability

HRV was measured using a Polar RS800 CX^®^ heart rate monitor and chest strap (Polar Electro OY, Kempele, Finland). Participants were instructed to empty their bladder before the measurement, which was conducted after a 5 min supine rest period with a stabilized HRV signal. The measurement consisted of a 5 min recording of RR intervals at a 1000 Hz sampling rate. Participants were instructed to breathe normally, remain quiet, and maintain a calm state throughout the measurement. Raw data were processed using the Elite HRV app (Elite HRV Inc, Asheville, United States), which has been validated for its validity and reliability^30^. The analysis of HRV included parameters such as the root mean square of successive differences between normal heartbeats (RMSSD), the standard deviation of all normal-to-normal intervals (SDNN), and HR.

#### Blood pressure

Systolic BP, diastolic BP, and PWV were obtained non-invasively using the Mobil-O-Graph^®^ (PWA-Monitor, IEM, Stollberg, Germany), which has been clinically validated as a reliable device for hemodynamic measurements^31^. Participants were instructed to rest in a supine position for 10 min prior to the measurement. At least two readings were taken on the right upper arm, using custom-fitted arm cuffs.

#### Cold pressor test

The cold pressor test is a common and extensively validated test for assessing cardiovascular reactivity. Trained study staff performed the cold pressor test before and after the intervention using a standardized protocol. Before the test, the participants rested in a supine position and had their BP, PWV, and HR measured. Subsequently, they immersed their right hand in cold water (5.0 °C) for two minutes while BP, PWV, and HR were monitored using the Mobil-O-Graph device. Cardiovascular reactivity was determined as the changes (Δ) in the respective parameters between the rest measurement and the subsequent cold pressor test. A numerical rating scale was used to assess participant’s level of pain on a scale ranging from 0 to 10, with 0 indicating no experience of pain and 10 indicating the most intense experience of pain possible.

#### Perceived stress

The Perceived Stress Scale (PSS) by Cohen et al.^32^ is a widely used tool for evaluating the perceived stress levels in individuals. The German version of the PSS-10^33^ was used in this study to assess the extent to which life events in the past week were perceived as unpredictable, uncontrollable, and overwhelming. The PSS-10 consists of 10 items rated on a 5-point scale (0 = “never” to 4 = “very often”). The PSS-10 has been established as reliable (Cronbach alpha = 0.84). The total score was calculated by summing up all 10 items, with higher scores indicating a higher level of perceived stress.

#### Affect

The Positive and Negative Affect Schedule (PANAS)^34^ is a widely used adjective-based questionnaire for assessing affective states, or emotions. The PANAS comprises 10 items measuring positive affect (PANAS+) and 10 items measuring negative affect (PANAS−). The German version of the PANAS was used in the present study, which has been validated as a reliable and valid tool with high internal consistency (Cronbach alpha = 0.84)^35^.

#### Vitality

The Subjective Vitality Scale (SVS)^36^ is a psychological measure used to assess an individual's sense of energy and aliveness. The SVS consists of a series of statements or questions related to the individual's experience of energy and engagement in life. The SVS consists of two scales, one referring to people’s general subjective vitality trait (SVSt) and a state-level scale, referring to subjective vitality at a specific moment (SVSs). The two scales have parallel items with few deviations that clarify the respective consideration level (i.e., in general versus at a specific moment). The participant rates their agreement with each statement on a 7-point rating scale, ranging from 1 (not at all true) to 7 (very true). High scores on the SVS are associated with greater psychological well-being, physical health, and life satisfaction, while low scores are related to symptoms of depression, anxiety, and other negative mental health outcomes. The SVS provides a quick and easy way to assess subjective vitality. The German 6-item Version (SVS-G) was used, which has been validated and shows a sufficient internal consistency (Cronbach alpha > 0.80)^37^.

### Intervention

#### Wim Hof method

The intervention followed the guidelines provided by Wim Hof^13^. The intervention comprised three components: cold water exposure, breathing exercise, and meditation. According to Hof^13^ positive changes of the WHM should already manifest themselves within 10 days. Thus, the participants were instructed to perform the WHM daily over the course of 15 days. The participants could choose when to do so throughout the day, however, the components were to be performed in a strict, sequential order: breathing exercises, followed by meditation, and then cold exposure, and at least 3 h postprandial. Participants were asked to complete a daily log in which they documented whether they performed the procedure and precisely which components they executed. Altogether, the procedure lasted about 15 min.

At the end of the baseline assessments, the participants received oral step-by-step instructions from the study staff, who were experienced in the WHM, on how to perform the various intervention components. They also received written instructions providing a step-by-step overview of the procedure, along with an audio file for the shower and meditation, and a video file for the breathing exercise.

#### Breathing-method

The breathing exercises consisted of a specific sequence of deep breaths in, holds, and breaths out, performed in a cyclic manner. The participants were provided with guided audio instructions. Participants were instructed to sit or lay in a comfortable position and perform 30 to 40 conscious breaths, inhaling deeply into their abdomen and chest and then exhaling without effort. After the last exhalation, they were instructed to hold their breath as long as they felt comfortable, then inhale deeply and hold their breath for 10 to 15 s. Thereafter, they exhaled again and relaxed. This was repeated two to three times.

#### Meditation

The meditation component of the WHM is a mindfulness-based technique that aims to regulate emotions and thoughts. Participants were instructed to find a quiet place, sit, or lie down in a comfortable position and focus their attention on their breathing and their body sensations. They were encouraged to pay attention to their thoughts, emotions, and bodily sensations and to bring their focus back to their breath if they become distracted. The meditation lasted 5 min or until the participant felt calm and centered. The goal was to cultivate a sense of inner peace, reduce stress and increase overall well-being.

#### Cold-water exposure

The cold-water exposure component involved exposure to cold water in the form of cold showers. Participants were instructed to take shower in cold water for a set period of time, starting with 30 s, and increase the time each day by 5 s as they become acclimated. The participants received an MP3 file that emitted a sound every ten seconds to keep track of the time. Furthermore, clear instructions were provided to the participants regarding the duration and specific body parts to be exposed to the cold. They began by showering their feet and legs (front and back) for 10 s, then their torso (front and back) for 10 s, and finally their entire body, including their head, for 10 s or the remaining time. The water temperature should be adjusted to the coldest possible setting.

### Statistics

Data analysis was conducted using IBM SPSS Statistics for Windows, Version 27.0 (IBM Corp., Armonk, NY, USA). The normal distribution of the data was checked using the Kolmogorov–Smirnov test. Independent samples t-test was applied to examine differences in baseline measures between the groups. To analyze the effect of time and group on the outcomes, a mixed-design analysis of variance (ANOVA) was employed. Post-hoc analyses, as appropriate, were performed using one-way ANOVA with Bonferroni correction. A significance level of *p* < 0.05 was used to determine statistical significance. The effect size was calculated using partial eta squared (*η*_*p*_^2^) and was categorized as small (0.01 ≤ *η*_*p*_^2^ < 0.06), medium (0.06 ≤ *η*_*p*_^2^ < 0.14), and large (*η*_*p*_^2^ ≥ 0.14)^38^.

## Results

One participant in the IG dropped out due to illness. In total, 21 participants in the IG and 20 participants in the CG were included in the final analysis. The age range was between 18 and 39 years. No adverse events occurred during the intervention period in any of the participants. The subject’s characteristics are displayed in Table 1. According to the BMI, four participants (IG = 1 and CG = 3) could be classified as overweight. According to WHtR one participant in the CG was classified as overweight.

**Table 1:** Participant’s characteristics at baseline.

	Total (*n* = 41)	IG (*n* = 21)	CG (*n* = 20)	*p* value
Age	26.7 ± 5.8	26.5 ± 5.9	26.9 ± 5.8	0.797
Height (cm)	179.9 ± 6.8	180.3 ± 5.9	179.6 ± 7.7	0.750
Body mass (kg)	73.8 ± 7.6	71.9 ± 7.8	75.7 ± 7.0	0.109
BMI (kg/m^2^)	22.79 ± 1.98	22.1 ± 1.8	23.5 ± 1.9	0.210
Waist-to-height ratio	0.45 ± 0.03	0.44 ± 0.02	0.45 ± 0.03	0.060

Data expressed as means ± standard deviations. *p* values indicate the differences between the intervention (IG) and control group (CG).

Based on the BP, eight participants were classified as having a high-normal BP (IG = 5, CG = 3) and two as hypertensive (IG = 1, CG = 1). According to the intervention logs the participants performed 13 ± 2 cold shower sessions, 12 ± 3 meditation sessions, and 12 ± 2 breathing exercise sessions throughout the 15-day intervention period. There were no significant time × group interactions (F(1,39) = 0.048, *p* = 0.828, *η*_*p*_^2^ = 0.001), and significant time effect F(1,39) = 1.848, *p* = 0.182, *η*_*p*_^2^ = 0.046) for MET hours.

### Cardiovascular parameters

No significant time × group interactions could be detected in HR (F(1,39) = 0.14, *p* = 0.709, *η*_*p*_^2^ = 0.004); RMSSD (F(1,39) = 0.05, *p* = 0.820, *η*_*p*_^2^ = 0.001), SDNN (F(1,39) = 1.82,* p* = 0.186, *η*_*p*_^2^ = 0.044), systolic BP (F(1,39) = 0.04, *p* = 0.839, *η*_*p*_^2^ = 0.001), diastolic BP (F(1,39) = 1.02, *p* = 0.318, *η*_*p*_^2^ = 0.026), and PWV (F(1,39) < 0.001, *p* = 0.983, *η*_*p*_^2^ < 0.001) (Table 2).

**Table 2:** Changes in cardiovascular outcomes, perceived pain, and MET hours from before to after intervention for the intervention group (IG) and control group (CG).

Outcome	IG (*n* = 21)		CG (*n* = 20)		*p* values	*η*_*p*_^2^
	Pre	Post	Pre	Post		
HR (min^−1^)	63.1 ± 10.3	62.8 ± 13.2	62.8 ± 13.1	61.8 ± 10.6	0.709	0.004
RMSSD (ms)	51.35 ± 38.55	53.42 ± 35.97	69.62 ± 33.83	70.25 ± 33.76	0.820	0.001
SDNN (ms)	64.39 ± 21.26	72.40 ± 34.49	83.20 ± 30.29	81.06 ± 29.58	0.186	0.044
SBP (mmHg)	123 ± 10	121 ± 10	120 ± 10	119 ± 8	0.839	0.001
DBP (mmHg)	72 ± 9	71 ± 7	69 ± 6	69 ± 7	0.318	0.026
PWV (m/s)	5.57 ± 0.52	5.54 ± 0.55	5.49 ± 0.63	5.45 ± 0.44	0.983	< 0.001
ΔHR (min^−1^)	3.5 ± 6.8	3.1 ± 6.1	3.8 ± 4.8	4.3 ± 5.7	0.596	< 0.001
ΔSBP (mmHg)	18 ± 12	18 ± 13	23 ± 10	17 ± 16	0.366	0.023
ΔDBP (mmHg)	18 ± 12	18 ± 12	19 ± 23	19 ± 14	0.999	< 0.001
ΔPWV (m/s)	0.29 ± 0.58	0.17 ± 0.61	0.43 ± 0.36	0.18 ± 0.59	0.635	0.007
Perceived pain	5.65 ± 1.73	4.60 ± 1.45	5.75 ± 1.94	5.65 ± 1.86	0.231	0.038
MET h	80.5 ± 55.8	68.6 ± 41.7	99.9 ± 80.9	83.5 ± 93	0.346	0.024

Data expressed as means ± standard deviations. *p* values represent interaction effects. *Pre* before intervention, *Post* after intervention, *HR* heart rate, *RMSSD* root mean sum of squared distance, *SDNN* standard deviation of RR-intervals, *SBP* systolic blood pressure, *DBP* diastolic blood pressure, *PWV* pulse wave velocity, *MET h* metabolic equivalent of task hours, *Δ* change in respective parameter from rest to cold pressor test, *η*_*p*_^2^ partial eta squared.

No main effects for time could be detected in HR (F(1,39) = 0.36, *p* = 0.554, *η*_*p*_^2^ = 0.009), RMSSD (F(1,39) = 0.18,* p* = 0.672, *η*_*p*_^2^ = 0.001), SDNN (F(1,39) = 0.61, *p* = 0.442, *η*_*p*_^2^ = 0.015; *p* = 0.656, *η*_*p*_^2^ = 0.005), systolic BP (F(1,39) = 1.39, *p* = 0.245, *η*_*p*_^2^ = 0.034), diastolic BP (F(1,39) = 0.22, *p* = 0.640, *η*_*p*_^2^ = 0.006), and PWV (F(1,39) = 0.24, *p* = 0.624, *η*_*p*_^2^ = 0.006).

Similarly, no main effects for group could be detected for HR (F(1,39) = 0.05,* p* = 0.843, *η*_*p*_^2^ = 0.001), RMSS (F(1,39) = 2.70, *p* = 0.108, *η*_*p*_^2^ = 0.065), SDNN (F(1,39) = 2.71, *p* = 0.108, *η*_*p*_^2^ = 0.065), systolic BP (F(1,39) = 1.03, *p* = 0.316, *η*_*p*_^2^ = 0.026), diastolic BP (F(1,39) = 1.27, *p* = 0.267, *η*_*p*_^2^ = 0.032), and PWV (F(1,39) = 0.33, *p* = 0.567, *η*_*p*_^2^ = 0.008).

### Cold pressor test

No significant time × group interactions could be detected in the stress response to the cold pressor test in ΔHR (F(1,39) = 0.29, *p* = 0.596, *η*_*p*_^2^ = 0.007), Δsystolic BP (F(1,36) = 0.84, *p* = 0.366, *η*_*p*_^2^ = 0.023), Δdiastolic BP (F(1,37) < 0.001, *p* = 0.999, *η*_*p*_^2^ < 0.001), ΔPWV (F(1,36) = 0.229, *p* = 0.635, *η*_*p*_^2^ = 0.007), and perceived pain (F(1,38) = 1.48, *p* = 0.231, *η*_*p*_^2^ = 0.038) (Table 2).

Again no main effect for time could be detected in ΔHR (F(1,39) = 0.002, *p* = 0.967, *η*_*p*_^2^ < 0.001), Δsystolic BP (F(1,36) = 0.89, *p* = 0.351, *η*_*p*_^2^ = 0.024), Δdiastolic BP (F(1,37) = 0.001, *p* = 0.973,* η*_*p*_^2^ < 0.0001), ΔPWV (F(1,36) = 2.007, *p* = 0.166, *η*_*p*_^2^ = 0.057), and perceived pain (F(1,38) = 2.17, *p* = 0.149, *η*_*p*_^2^ = 0.054). Similarly no main effect for group could be detected for ΔHR (F(1,39) = 0.24, *p* = 0.630, *η*_*p*_^2^ = 0.006), Δsystolic BP (F(1,36) = 0.82, *p* = 0.371, *η*_*p*_^2^ = 0.022), Δdiastolic BP (F(1,37) = 0.03, *p* = 0.855, *η*_*p*_^2^ = 0.001), ΔPWV (F(1,36) = 0.368, *p* = 0.548, *η*_*p*_^2^ = 0.011), and perceived pain (F(1,38) = 2.14, *p* = 0.152, *η*_*p*_^2^ = 0.053).

### Psychological parameters

There were no significant time × group interactions for  PANAS+ (F(1,38) = 0.65, *p* = 0.427,* η*_*p*_^2^ = 0.017), PANAS− (F(1,38) = 0.26, *p* = 0.614, *η*_*p*_^2^ = 0.007), SVSt (F(1,38) = 0.09, *p* = 0.760, *η*_*p*_^2^ = 0.002), SVSs (F(1,38) = 0.84, *p* = 0.366, *η*_*p*_^2^ = 0.022), and PSS (F(1,38) = 0.84, *p* = 0.364, *η*_*p*_^2^ = 0.022) (Table 3).

**Table 3:** Changes in psychological outcomes from before to after intervention for the intervention group (IG) and control group (CG).

Outcome	IG (*n* = 21)		CG (*n* = 20)		*p* values	*η*_*p*_^2^
	Pre	Post	Pre	Post		
PANAS+	3.23 ± 0.33	3.19 ± 0.29	3.32 ± 0.30	3.14 ± 0.44	0.427	0.017
PANAS−	1.82 ± 0.60	1.85 ± 0.37	1.75 ± 0.47	1.69 ± 0.54	0.614	0.007
SVSt	5.02 ± 0.94	5.04 ± 0.92	5.02 ± 1.0	5.11 ± 0.99	0.760	0.002
SVSs	4.79 ± 0.98	5.12 ± 0.90	4.70 ± 1.00	4.68 ± 1.13	0.366	0.022
PSS	16.0 ± 4.6	14.3 ± 4.0	17.2 ± 5.0	14.3 ± 4.2	0.364	0.022

Data expressed as means ± standard deviations. *p* values represent interaction effects. *Pre* before intervention, *Post* after intervention, *PANAS*+ positive affect, *PANAS*− negative affect, *SVSt* subjective vitality trait, *SVSs* subjective vitality state, *PSS* perceived stress, *η*_*p*_^2^ partial eta squared.

Only the PSS revealed a significant main effect for time (F(1,38) = 10.94, *p* = 0.002, *η*_*p*_^2^ = 0.224). For all the other parameters, there was no effect for time (PANAS+ : F(1,38) = 1.99, *p* = 0.166, *η*_*p*_^2^ = 0.050; PANAS−: F(1,38) = 0.03,* p* = 0.864, *η*_*p*_^2^ = 0.001; SVSt: F(1,38) = 0.20, *p* = 0.660, *η*_*p*_^2^ = 0.005; SVSs: F(1,38) = 0.68,* p* = 0.416, *η*_*p*_^2^ = 0.017).

Furthermore, no significant group effects could be revealed for PANAS+ (F(1,38) = 0.07, *p* = 0.799, *η*_*p*_^2^ = 0.002), PANAS− (F(1,38) = 0.738, *p* = 0.396, *η*_*p*_^2^ = 0.019), SVSt (F(1,38) = 0.01, *p* = 0.916, *η*_*p*_^2^ < 0.001), SVSs (F(1,38) = 1.06, *p* = 0.310, *η*_*p*_^2^ = 0.027), and PSS (F(1,38) = 0.22, *p* = 0.642, *η*_*p*_^2^ = 0.006).

## Discussion

The present study aimed to determine the effectiveness of the WHM on various psychological and physiological parameters. No significant time × group interaction could be detected for any of the outcomes. Thus, contrary to the claim by Wim Hof^13^, performing the WHM on a daily basis for 15 days did not exert positive effects on cardiovascular parameters, perceived stress, affect, and vitality in this study. Moreover, the cardiovascular stress response and perceived pain during a cold pressure test were not affected by regularly performing the WHM.

### Cardiovascular parameters

Unfortunately, there are no comparable studies assessing the effects of the WHM on cardiovascular parameters. However, previous studies on the different components of the WHM can inform the interpretation of the results.

It has been often proposed that regular cold exposure may possibly comprise a healthier cardiovascular risk profile, as cold exposure triggers a cascade of physiological reactions, eventually leading to an increased parasympathetic and a blunted sympathetic activation^3,21,39^. However, our study found no significant changes in HR, HRV parameters, BP, and PWV after regularly performing the WHM. Previous results on the effects of cold exposure on cardiovascular parameters report conflicting result. Michalsen et al.^40^ found that resting HR and rate-pressure product were significantly lower after a 6 weeks hydrotherapeutic program in patients with mild chronic heart failure.

In contrast to our study, the hydrotherapeutic program included not only cold applications but also peripheral warm water baths, such as arm baths and foot baths. Mäkinen and colleagues^21^ demonstrated a significant increase in parasympathetic activity and a decrease in systolic and diastolic BP following exposure to cold air for 10 days. In this study, the duration of cold exposure was 2 h, which was significantly longer than in our study. Likewise, Jacob et al.^41^ reported a significant reduction in systolic and diastolic BP in hypertensive patients following a 19 days multidisciplinary cardiac rehabilitation program that included hydrotherapy, in comparison to a control group that received the multidisciplinary cardiac rehabilitation treatment without hydrotherapy. On the contrary, Hirvonen et al.^42^ reported no significant changes in BP in winter swimmers compared to a control group. Similarly, Westerlund et al.^43^ found no significant changes in resting BP after three months of whole-body cryotherapy in healthy, normotensive men and women. The intervention consisted of three cryotherapy sessions per week in a therapy chamber with temperatures ranging from − 110 to − 113 °C.

The discrepancies may be attributed to differences in study design, such as the form of exposure (cryotherapy, whole-body cold-water immersion, cold shower), the exposure duration, the body area exposed to cold, the temperature, and the sessions’ frequency^44^. Additionally, the participants' characteristics may also influence the results. It seems that especially risk patients, benefit from cold water immersion, as reported in the study from Michalsen et al.^40^ and Jacob et al.^41^, but not healthy individuals as included in the present study. It is worth noting that some forms of cold exposure included physical activity like swimming and water treading. Therefore, possible covariance of physical activity in these studies can’t be ruled out.

### Cardiovascular reactivity

Previous research has demonstrated that cold water immersion stimulates positive adaptive changes in the human body, resulting in increased readiness to handle stress factors^3^. These changes may be attributed to a reduction in sympathetic nervous system reactivity, as well as increased antioxidative protection^3^. According to Jacob et al.^41^, repeated vasomotor stimulation through repeated cold-water exposure leads to a down-regulation of adrenergic receptors and thus to a reduced sensitivity of the resistance vessels to catecholamines. Based on this, we hypothesized that regular cold exposure through the WHM could reduce cardiovascular reactivity to the cold pressor test and a reduction in perceived pain. The cold pressor test consists of a pain and cold component that activates the sympathetic nervous system, leading to significant α-adrenergic vasoconstriction, increased total peripheral resistance, and subsequent elevations in BP^45^.

We postulated that regular cold exposure could mitigate the vasoconstrictive effects of acute cold exposure, thereby reducing the cardiovascular response to the cold pressor test. This would be of relevance as the magnitude of an individual’s hemodynamic responses to a stressor can be a marker for cardiovascular risk^46^.

Previous research has shown that repeated exposure to cold environments decreases sympathetic responses to acute cold exposure, as evidenced by reduced changes in HRV, BP, and plasma norepinephrine^21^.

However, in the present study, no significant changes in HR, BP, PWV, and perceived pain could be reported after the WHM. As mentioned earlier, the study by Mäkinen et al.^21^ employed a significantly longer cold exposure duration (2 h), which could have triggered more robust acute reactions, ultimately leading to more pronounced adaptive processes in the long term. Our results are in line with Louis et al.^44^, who found that HRV indices and BP changes didn’t alter between 1 and 5 sessions of whole-body cryotherapy. Furthermore, Leppäluoto et al.^39^ found that cold-induced stimulation of plasma norepinephrine remained at the same levels throughout 12 weeks of winter swimming or whole-body cryotherapy. These conflicting results may again be explained by differences in study design and study participants. Furthermore, it should be noted that prior research on cardiovascular reactivity has typically examined cold exposure as a standalone intervention, unlike our study, where it was combined with other intervention components. It could be speculated that these additional components (such as breathing exercises and meditation) might have mitigated acute physiological reactions by promoting better mental readiness for the stress-inducing situation. Further studies are warranted to investigate the potential compromising effects of the different components of the WHM.

### Meditation

Besides cold-water immersion, also meditation techniques have been reported to lower BP and improve HRV in older or at-risk patients^2,47^. Meditation is believed to reduce stress and physiological arousal, leading to favorable effects on the balance of the autonomic nervous system^47^. However, the literature on the effects of meditation on cardiovascular risk factors in healthy individuals is less consistent. A Cochrane review was unable to draw any conclusions on the effectiveness of meditation due to considerable variations between studies^48^.

In the present study, the WHM, which includes a meditation component, could not induce changes in HR, HRV, BP, and PWV. It's worth noting that the intervention period in the present study was relatively short compared to that in other studies, many of which had durations exceeding 2 months^2,48^. However, a study by Nidich et al.^49^ also failed to show any changes in BP after a 3 months meditation intervention in a cohort of individuals with a similar age and normal BP.

### Breathing

Slow deep breathing, commonly practiced in meditation, yoga, and several relaxation techniques, has been shown to positively affect BP^4^. The breathing exercise applied in the WHM is linked to Tummo Meditation, which involves both somatic and neurocognitive components that lead to hyperventilation. This technique is not characterized by relaxation, but rather by a state of arousal induced by hyperventilation and hypoxia resulting from breath retention. This has been shown to affect the sympathetic nervous system by increasing catecholamines^50^.

Recent studies by Zwaag et al.^16^ and Kox et al.^15^ have shown that the WHM breathing exercise, which results in intermittent respiratory alkalosis and hypoxia, increases plasma catecholamines and causes an increase in HR and a decrease in mean arterial pressure.

Unfortunately, there are no studies on the long-term effects of this breathing technique on HR, HRV, BP, and PWV. According to the results of the present study, regularly performing the breathing exercises in combination with cold water exposure and meditation did not alter HRV and hemodynamic parameters.

### Psychological parameters

The present study did not detect any significant changes in affect (positive and negative), vitality, and perceived stress after regularly performing the WHM.

To the best of our knowledge, no previous studies have addressed the effects of regularly performing the WHM on these psychological outcomes. Only, Petraskova et al.^14^ reported that an 8 weeks WHM training program for healthy participants during an Antarctic expedition significantly reduced depressive symptoms in comparison with a control group. However, there is a significant body of literature showing that the components of the WHM can positively affect the respective parameters.

In the study by Huttunen et al.^9^, winter swimmers claimed to be more energetic, active, and brisk than the controls. The authors also found that winter swimming increased mood and decreased fatigue and tension, which may be linked to an enhanced secretion of catecholamines^9^. However, this study utilized other questionnaires (Profile of Mood States) compared to our study. In another study by Lubkowska^51^, winter swimmers believe that cold stimulation improves their ability to cope with daily stress.

Meditation has also demonstrated promising results in reducing psychological distress across different populations and settings and can be effective in enhancing vitality^52^. However, according to a recent meta-analysis, the positive effects of meditation interventions were only evident in at-risk populations, such as those experiencing stressful life situations^53^. This may help explain why we were unable to detect any changes in our participants, who were not inherently in stressful life situations^32^.

It is unclear why the present intervention did not induce positive psychological effects. However, it is possible that the considerable differences in interventions applied, and participants included may account for the differences. Furthermore, the intervention period of previous studies was considerably longer. It can be speculated that longer interventions may be necessary to produce more substantial changes in psychological parameters. Moreover, it cannot be ruled out that the combination of the different components of the WHM may have blunted the effects of each other. Further research is needed to determine the effects of regularly performing the WHM on psychological outcomes.

### Limitations

Several limitations of this study must be addressed. First, the study only included healthy young male adults, which may limit the generalizability of the results to other populations, such as individuals with a higher risk profile or female participants. Second, the home-based intervention did not allow for standardization of water temperature. Even though the participants were instructed to set the water temperature as low as possible, there were considerable differences in the water temperature throughout the intervention period and in the different regions where the participants lived. According to the local water provider (Wasserverbund Region Bern AG), the mean water temperature throughout the intervention period was 14.1 ± 3.2 °C.

Third, although participants received clear instructions on the duration and the body parts to be exposed to the cold water, compliance with these instructions was only documented using a daily log. Fourth, the intervention duration was relatively short. Even though Wim Hof claims that changes will occur after 10 days, it is expected that more extended intervention periods lead to more pronounced effects. Fifth, it would be interesting to investigate the individual effects of different combinations of the intervention components (hyperventilation, meditation, cold exposure) before exploring their combined effects. Sixth, we only included individuals with no prior experience with the various components of the intervention. It could be argued that, especially for the meditation component, some prior experience and a specific mindset might be beneficial.

Finally, it is unclear whether it is necessary to be trained by the creator of the intervention or a trained instructor with regard to the so-called guru effect^54^. However, Zwaag et al.^16^ found that the effects of the intervention were independent of the individual who provided it. Furthermore, the creator claims that the WHM is a method which can be performed independently at home^13^.

## Conclusion

The WHM has gained popularity for its potential health and performance benefits, including performance enhancement, improved mood, reduced stress, enhanced immune function, improved cardiovascular function, and increased vitality^13^. Despite its growing popularity, there is limited scientific evidence to support these claims. This is the first study assessing the effects of the WHM on cardiovascular parameters. According to the presented results, the positive effects of the WHM on cardiovascular and psychological parameters, as often claimed, cannot be confirmed. In a group of healthy individuals, the short-term application of this method did not exert any positive effects. Based on the present results, the WHM should be questioned as a complementary therapy in cardiovascular prevention.

Further studies should address the effects of a longer and more intense intervention, and should include additional parameters, especially non-linear HRV parameters, which can aid in a better interpretation of the physiological behavior of HRV. Furthermore, studies should consider a dismantling design in which the different components are compared to the full protocol. This could help determine possible opposing effects on physiological and psychological parameters.

## Data Availability

All raw data are available on request to the corresponding author.

## Abbreviations

ANOVA: Analysis of variance
BP: Blood pressure
BMI: Body mass index
CG: Control group
CVD: Cardiovascular diseases
HR: Heart rate
HRV: Heart rate variability
IG: Intervention group
MET: Metabolic equivalent of task
PANAS: Positive and negative affect schedule
PSS: Perceived stress scale
PWV: Pulse wave velocity
RMSSD: Root mean square of successive differences between normal heartbeats
SDNN: Standard deviation of all normal-to-normal intervals
SVS: Subjective vitality scale
SVSs: Subjective vitality scale state
SVSt: Subjective vitality scale trait
WHM: Wim Hof Method
WHtR: Waist-to-height ratio
